# Rigid-body motion is the main source of diffuse scattering in protein crystallography

**DOI:** 10.1107/S2052252519000927

**Published:** 2019-02-16

**Authors:** T. de Klijn, A. M. M. Schreurs, L. M. J. Kroon-Batenburg

**Affiliations:** a Crystal and Structural Chemistry, Bijvoet Center for Biomolecular Research, Utrecht University, Padualaan 8, 3584 CH Utrecht, The Netherlands

**Keywords:** diffuse scattering, protein crystallography, rigid-body motion, protein dynamics

## Abstract

The origin of diffuse X-ray scattering from protein crystals has been the subject of debate over the past three decades regarding whether it arises from correlated atomic motions within the molecule or from rigid-body disorder. Here, a supercell approach to modelling diffuse scattering is presented that uses ensembles of molecular models representing rigid-body motions as well as internal motions as obtained from ensemble refinement. This approach allows oversampling of Miller indices and comparison with equally oversampled diffuse data, thus allowing the maximum information to be extracted from experiments. It is found that most of the diffuse scattering comes from correlated motions within the unit cell, with only a minor contribution from longer-range correlated displacements. Rigid-body motions, and in particular rigid-body translations, make by far the most dominant contribution to the diffuse scattering, and internal motions give only a modest addition. This suggests that modelling biologically relevant protein dynamics from diffuse scattering may present an even larger challenge than was thought.

## Abstract 2

Diffuse scattering is caused by correlated motions in protein crystals and is a potential source of information on protein dynamics. Although internal motional models were able to reproduce the diffuse scattering to a limited extent in earlier research, it is shown here that by far the most dominant contribution is from rigid-body translations, with internal motions contributing only a small part to the total scattering. Possibilities for extracting information on internal motions, despite these findings, are discussed.

## 1. Introduction   

X-ray crystallography has been the main method for solving macromolecular structures for several decades. With the advent of highly brilliant X-ray sources and photon-counting pixel-array detectors, it has evolved into a highly automated technique, even for very small micrometre-sized crystals of large molecular complexes; this has allowed its widespread use by structural biologists. Crystallography makes use of the enhancement of X-ray scattering caused by the periodic arrangement of molecules in a lattice, and data-collection and structure-solution techniques focus on obtaining the intensities of the Bragg reflections and using them to refine a structural model. Any background scattering is removed in the integration process and is treated as a nuisance rather than as a carrier of information. However, correlated motion or disorder of atoms in the crystal causes diffuse scattering outside the Bragg peaks (note that X-ray diffraction experiments cannot distiguish between static and dynamic disorder). While amplitudes of motion result in the *B* factors, it is the correlation in motion that is exclusively contained in the diffuse scattering. It is estimated that for a protein crystal with a modest *B* factor of 20 Å^2^ the total diffuse scattered intensity exceeds that of the Bragg intensity beyond a resolution of 3.8 Å (Clarage *et al.*, 1992 ▸). Access to information on correlated motion of biomolecules could provide insight into their dynamics, which are generally considered to be crucial to their function (Henzler-Wildman & Kern, 2007 ▸). Understanding and modelling the diffuse scattering potentially adds valuable information to what we can learn from Bragg scattering (Meisburger *et al.*, 2017 ▸).

The first attempts at interpreting diffuse scattering in terms of protein-molecule motions or internal mobilities were made in the 1980s and 1990s. In a seminal paper, Caspar *et al.* (1988 ▸) developed a liquid-like model to explain the observed variational diffuse scattering features of rhombohedral insulin crystals (see Section 2 for a description of the various types of diffuse scattering). They found that the two main features that were observed, broad cloudy scattering and narrower halos around the Bragg peaks, could be modelled by two displacement correlation functions with coupling distances of 6 and 20–30 Å, respectively. They ruled out the possibility that the diffuse scattering was caused by low-frequency lattice vibrations which would give rise to thermal diffuse scattering (TDS), as these would produce much narrower halos. In contrast, their observations indicated significant correlation between nearest-neighbour molecules. In a later paper by Clarage *et al.* (1992 ▸), this approach was further extended and applied to triclinic and tetragonal lysozyme crystals. Again, for each crystal two components of the diffuse scattering could be modelled: a short-range correlation of internal movements with a coupling distance of 6 Å, which was interpreted as changes of torsion angles in the backbone or neighbouring side-chain displacements, and long-range lattice-coupled displacements of 50 Å in distance. In contrast to these findings, Pérez *et al.* (1996 ▸) concluded that rigid-body movements are the major contribution to the diffuse scattering of tetragonal lysozyme crystals. Their model reproduced the shape of the observed diffuse patches (speckles) with roughly equal contributions from translational and rotational displacements. A further argument for this model is that the *B* factors of C^α^ positions are reproduced. Molecular-dynamics simulations of orthorhombic lysozyme (Héry *et al.*, 1998 ▸) further supported rigid-body translations, although it was suggested that only the backbone atoms form the rigid core, with the side chains forming separate rigid bodies.

In the following years, Wall and coworkers (Wall, Clarage *et al.*, 1997 ▸; Wall, Ealick *et al.*, 1997 ▸) published methods to extract three-dimensional diffuse scattering maps from experimental data. Until then, all data had been extracted from single (still) images and mapped onto the two-dimensional detector plane by intersection with the Ewald sphere. They applied their techniques to staphylococcal nuclease and calmodulin crystals and fitted the diffuse scattering in both cases using Caspar’s liquid-like motional models, although in the latter case there were additional streaks in the scattering data caused by nearest-neighbour coupling that required an anisotropic treatment.

The debate on whether the variational diffuse scattering is caused by internal correlated motion or rigid-body translations and rotations became dormant for some time, but has recently been revived, starting with a series of papers by Van Benschoten and Wall (Van Benschoten *et al.*, 2015 ▸, 2016 ▸; Wall, 2018 ▸). In the first paper, diffuse scattering maps are generated from translation–libration–screw (TLS) models as used in the structural refinement of protein crystal structures. However, different selections of TLS groups produced markedly different diffuse patterns. In a very enlightening paper, Van Benschoten *et al.* (2016 ▸) showed that three-dimensional diffuse scattering data can be obtained from routine data collections from protein crystals using the highly brilliant X-ray sources that are currently available and modern pixel-array detectors (PADs). They analysed the diffuse scattering of cyclophilin A (CypA) and trypsin using various models and concluded that TLS models did not agree well with the data, but that normal-mode (NM) analysis and liquid-like motion (LLM) models gave much better agreement. In contrast, Ayyer *et al.* (2016 ▸) concluded that the continuous scattering visible as a speckle pattern in XFEL data beyond the 4.5 Å Bragg limit from crystals of the integral membrane-protein complex photosystem II is caused by translational lattice disorder. The diffuse scattering then becomes the incoherent sum of many (rotationally) aligned single-molecule diffraction patterns. Iterative phasing of the continuous diffraction gave Fourier amplitudes and phases to 3.3 Å resolution and much-improved electron density. This method is further detailed in Chapman *et al.* (2017 ▸). Recently, Peck and coworkers showed evidence for longer-range intermolecular correlated motions, *i.e.* longer than the size of one molecule, in three different protein crystals (Peck *et al.*, 2018 ▸), and Polikanov and Moore suggested displacements arising from acoustic lattice vibrations in ribosome crystals, implying low-frequency motions of whole molecules (Polikanov & Moore, 2015 ▸). Previously, this long-range order had also been observed by Doucet & Benoit (1987 ▸) for orthorhombic lysozyme.

Models for diffuse scattering from protein crystals can be subdivided into those that use analytical expressions with only a few parameters, such as the liquid-like motion model, and those that use molecular model coordinates, such as normal-mode analysis, TLS models and molecular-dynamics simulations. None of these approaches has given a conclusive structural interpretation of the correlated motion that is responsible for diffuse scattering. A comprehensive review containing an excellent section on diffuse scattering can be found in Meisburger *et al.* (2017 ▸). The quality indicators that should be used to quantify the agreement between modelled and experimental diffuse data have not yet been well established in the field. For Bragg data, *R*_work_ and *R*_free_ in structural refinement and real-space electron-density correlation coefficients between model and observed data are well accepted.

In this work, we study how diffuse scattering is built up from various structural contributions in the full three-dimensional reciprocal space. We simulate diffuse scattering from an ensemble of molecular models that represent disorder in crystals through rigid-body motions and/or internal motions. For this, we sampled rigid-body translations and rotations from Gaussian distributions based on the refined *B*-factor fingerprint or sampled poses from motions described by TLS models, and generated ensembles from ensemble refinement of the crystal structures (Burnley *et al.*, 2012 ▸) to model internal motions. The diffuse maps were calculated by our newly developed supercell method, allowing sampling of reciprocal space in between integer Miller indices. We extracted diffuse scattering intensities from experimental diffraction data of CypA and lysozyme and converted these to full three-dimensional reciprocal-space maps. Since the diffuse signal is continuous through reciprocal space, sampling only on Bragg spots can lead to a loss of information. The size of the pixels and the rotation scan width of the images allows oversampling of the Miller indices by a factor of 5–10, *i.e.* 5^3^–10^3^ more voxels can be assigned than just those belonging to integer Miller indices. We will show that rigid-body contributions to diffuse scattering are dominant by analysing different aspects. (i) We calculate linear correlation coefficients (CCs) between the maps and compare these with literature values. (ii) We visually inspect intensity distributions (speckle patterns) in both the calculated and experimental two-dimensional and three-dimensional diffuse maps. (iii) We calculate the contribution of internal motion to the diffuse features. (iv) We make an unbiased estimate of the structural unit that is responsible for the diffuse scattering by calculating the Patterson map of the experimental diffuse data.

## 2. Theory of diffuse scattering from disordered crystals   

Diffuse scattering caused by static or dynamic disorder can be understood by considering the general equation for the total scattering of a crystal in terms of a lattice summation of unit cells containing the scattering atoms, 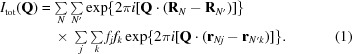



The first double summation is over all periodic lattice points with positional vectors **R**_*N*_ in three dimensions; the second term runs over the positional coordinates of atoms in the unit cells. **Q** is the vectorial difference between the incident and scattered wavevectors and has length 1/*d* = 2sinθ/λ. If the crystal were strictly ordered, the total diffracted intensity^1^ would be
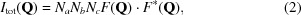
where *N_a_* is the number of unit cells along the *a* axis, and likewise for *N_b_* and *N_c_*, and *F* is the structure factor of every unit cell. Let us consider deviations of atoms from their ideal positions in the unit cells. Each atom *j* will be displaced by a vector **δ**_*j*_ from its average position 〈**r**_*j*_〉 . The total scattering then becomes
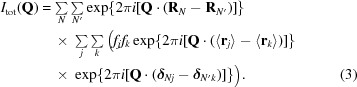



The variation of atom positions produces diffuse scattering and is dependent on the type of motion or disorder. Four classes can be distinguished.

(i) The weak spherical scattering around the incident beam caused by uncorrelated random displacements.
(ii) Broad cloud-like features between the Bragg peaks caused by correlated motions within the unit cell, and often called very diffuse or variational scattering (Caspar *et al.*, 1988 ▸). The correlation between atoms can either be the result of the internal flexibility of protein molecules, *e.g.* global variations in domain secondary structure or side-chain conformations, or be caused by rigid-body motions of entire molecules, or any combination of these.
(iii) Halos around the Bragg peaks caused by correlation over several unit cells.
(iv) Sharp features such as streaks, rings or triangles from long-range correlations.

(3) is the general equation for describing diffuse scattering and can be expanded in several ways. We follow James (1958 ▸) in deriving the results for random, independent and isotropic displacements of atoms. Averaging over the unit cells reduces the last exponential to exp{−2π^2^[(〈**δ**_*j*_ − **δ**_*k*_〉)·**Q**]^2^} (where use is made of a Taylor expansion, cut off after the quadratic term) and in addition 〈**δ**_*j*_ − **δ**_*k*_〉^2^ ≃ 〈**δ**_*j*_^2^〉 + 〈**δ**_*k*_^2^〉.

(3) then becomes
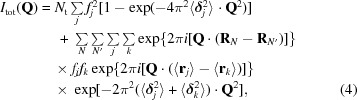
where *N*_t_ is the number of unit cells in the crystal. The last term is the usual Bragg intensity modulated with the Debye–Waller factor, and peaks at Miller indices because of the lattice sum. The first term is the diffuse scattering of type (i) that is spherical around the incident beam, and the reduction in intensity by the Debye–Waller factor from the Bragg part reappears in the diffuse scattering.

Now, suppose that the unit cell contains one molecule (*P*1 symmetry) and that the molecules have random isotropic translational displacements. The atomic displacements are thus fully correlated and all atoms within a unit cell are displaced over the same vector **δ**_*N*_. The subscripts *j* and *k* in (3) can be dropped and, following the same reasoning as above, we obtain 
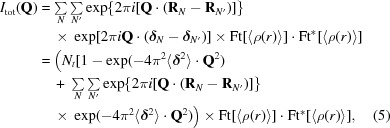
where Ft[〈ρ(*r*)〉] is the Fourier transform of the average electron density and 〈**δ**〉 is the average displacement. We see that the diffuse scattering is proportional to the squared Fourier transform of the unit-cell density. In the case of symmetry-related molecules that are displaced independently, Ayyer *et al.* (2016 ▸) have shown that the diffuse scattering is proportional to the incoherent sum of the squared Fourier transforms of the independent rigid units. This principle was exploited by Ayyer *et al.* (2016 ▸) and Chapman *et al.* (2017 ▸), who used continuous scattering from translationally disordered crystals for phasing beyond the Bragg diffraction limit. The diffuse scattering is that of type (ii). It is important to note that the maximum diffuse scattered intensity is achieved by these rigid-body translations as *all* atoms move in a correlated fashion and the Fourier transform of the *whole* molecule appears in (5). Also note that increasing the average displacement 〈**δ**〉 (*i.e.* increasing the disorder of the crystal) does not change the diffuse pattern (the Fourier transform) but only scales the intensties.

An effort to derive such equations to incorporate rotational disorder was undertaken by Moore (2009 ▸). It followed from his paper that the diffuse scattering caused by rotational disorder looks completely different from that of translation disorder. If atomic displacements are correlated in a complex way, including rigid-body rotations, it is easier to rearrange (3) by incorporating all atomic displacements into the varying structure factors (Welberry, 1985 ▸; Moss *et al.*, 2003 ▸), 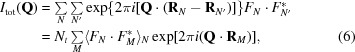
where **R**_*M*_ is the difference vector between unit-cell origins. (6) can be rewritten as 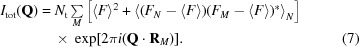



The first part is the Bragg scattering; the second part, which contains a possible correlation between unit cells **R**_*M*_ apart, is responsible for the diffuse scattering. When correlations exist between atom motions on length scales larger than the unit cell, sharp diffuse scattering of types (iii) and (iv) is observed. It is convenient to rewrite the second part of (7) in terms of correlation coefficients (Moss *et al.*, 2003 ▸). In this paper, we are only concerned with diffuse scattering of type (ii). Thus, if no correlations across unit cells exists, (7) reduces to
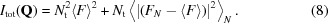



The first part is the Bragg scattering, which becomes *N*_t_〈*F*〉^2^ after integration over the peak width resulting from the finite size of the crystal. The second part is the diffuse scattered intensity and is commonly rewritten as
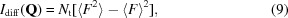
the well known Guinier equation for modelling diffuse scattering caused by motions within the unit cell and which we exploited in this work. Thus, for such motions it is sufficient to calculate the variance in structure factors.

## 3. Materials and methods   

### 3.1. Diffraction data for CypA and hen egg-white lysozyme   

Experimental data for cyclophilin A (CypA) were obtained from the SBGrid Data Bank (https://data.sbgrid.org/dataset/68; Fraser, 2015 ▸). The data were recorded on beamline 11-1 at Stanford Synchrotron Radiation Light source using a Dectris PILATUS 6M pixel-array detector, a rotation range of 180°, a rotation scan width of 0.5° and an exposure time of 0.2 s. The data were from a single crystal at an ambient temperature of 293 K with minimal surrounding mother liquor. The data were indexed with *DirAx* (Duisenberg, 1992 ▸); unit-cell and instrument parameters were refined with *Peakref* (Schreurs, 1999*b* ▸). A significant offset from the horizontal orientation of the spindle axis was found with some 5° of reorientation of the crystal during the scan. Refined unit-cell matrices were used for reciprocal-space reconstruction. The structural models were generated based on refinement by Van Benschoten *et al.* (2016 ▸) and deposited as PDB entry 5f66.

Crystals of hen egg-white lysozyme (Sigma–Aldrich, Schnelldorf, Germany) were obtained using the hanging-drop vapour-diffusion method with a protein concentration of 25 mg ml^−1^. The crystals had dimensions of 100 × 100 × 20 µm. Data were collected on beamline ID-30A-3 at the European Synchrotron Radiation Facility (ESRF) using a Dectris EIGER X 4M detector. One crystal was mounted on a MicroMesh Crystal Mount (MiTeGen) and kept at constant humidity using the HC1 Humidity Control Device (Sanchez-Weatherby *et al.*, 2009 ▸) and ambient temperature (293 K). Images were recorded over a rotation range of 180° and were fine-sliced in 0.1° per image with 0.01 s exposure. Images were merged into 1° frames prior to indexing with *DirAx*. The unit-cell matrix was refined with *Peakref* (Schreurs, 1999*b* ▸) and reflection data were processed with *EVAL*15 (Schreurs *et al.*, 2010 ▸) to 1.3 Å resolution (Supplementary Table S1) and scaled using *SADABS* (Sheldrick, 1996 ▸). The structure was refined against these data using *phenix.refine* (Adams *et al.*, 2010 ▸; Supplementary Table S1).

### 3.2. Reconstruction of diffuse scattering maps in reciprocal space   

**Figure 1:**
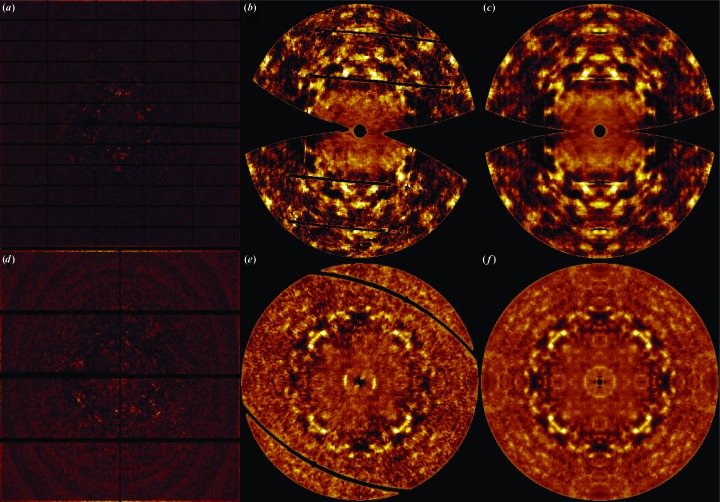
Experimental diffraction detector images for (*a*) CypA and (*d*) lysozyme after mode filtering and radial subtraction. The reconstructed *hk*0 reciprocal-space slice for CypA (*b*) after intensity merging with Friedel symmetry (−1) and (*c*) after merging with Laue symmetry (*mmm*). The *hk*0 reciprocal-space slice of lysozyme (*e*) after intensity merging with Friedel symmetry (−1) and (*f*) after merging with Laue symmetry (4/*mmm*). The slices comprise voxels between *l* = −1/5 and 1/5 and *l* = −1/10 and 1/10 for CypA and lysozyme, respectively. The slices range from *h* = −22 to 22, *k* = −27 to 27 for CypA and *h* = −40 to 40, *k* = −40 to 40 for lysozyme.

All of the software used to generate diffuse scattering maps forms part of the *EVAL* software suite (Adams *et al.*, 2010 ▸; Schreurs, 1999*a* ▸). For each image, bad-pixels masks were generated. These comprise panel gaps (indicated by a pixel value of ‘−1’ in the image file) and a user-defined beam-stop shadow. To remove parasitic scattering of air and solvent surrounding the crystal and inelastic Compton scattering, a circularly averaged profile was subtracted. This profile was constructed using pixels with values of less than 0.5 of the maximum pixel intensity in the image and was corrected for polarization of the synchrotron beam. When subtracting the radial profile the polarization was reintroduced. To isolate the diffuse scattering, Bragg spots had to be removed. Methods have been described in the literature that use knowledge of Bragg peak positions. Masks are located at predicted reflection positions and, within these, pixels are removed only if they deviate significantly from the background (Polikanov & Moore, 2015 ▸; Peck *et al.*, 2018 ▸). An alternative method that is not dependent on predicting reflection positions and that is often used to remove sharp features in images is mode filtering (Wall, Ealick *et al.*, 1997 ▸). The most common value of the pixel intensities in a box around every pixel replaces its value. We took this approach and investigated how well Bragg reflections were removed depending on the kernel size. We found that a kernel size of 21 × 21 pixels was needed to remove the Bragg spots completely. Background and Bragg peak removal is implemented in *VIEW* (Schreurs, 1999*a* ▸). Examples of the resulting images containing only diffuse scattering for CypA and lysozyme are shown in Fig. 1 ▸. Once the radial scattering and Bragg peaks have been removed, the pixels are transformed to reciprocal space by the software *IMG*2*HKL*, which is part of the *EVAL* package (Schreurs, 1999*a* ▸). In fact, every pixel represents a voxel extending in the rotation direction over the scan width. The eight corners are mapped to reciprocal space and the intensity is divided over the voxels that are touched in the new grid. We chose to define the new grid in terms of supercell (*h*_s_, *k*_s_, *l*_s_) indices for easy comparison with the simulated diffuse maps (see Section 3). The supercell indices correspond to rational fractions of Miller indices of the original unit cell. For CypA we used a 9 × 8 × 5 supercell, allowing sub-Miller-index sampling in multiples of 1/9, 1/8 and 1/5 in the a*, b* and c* directions, respectively. For lysozyme we used a 5 × 5 × 10 supercell. In both cases the target voxels represent roughly the same dimension in Å^−1^. The resolution limit of the pixel data we used was 2.0 Å in both cases. During the mapping, image voxel intensities are corrected for Lorentz and polarization factors and accumulated in the target voxels (*h*_s_, *k*_s_, *l*_s_). Thus, the final values are proportional to squared structure factors. However, a particular region in reciprocal space can occur twice in a rotation scan ranging over less than 360°: one time left and one time right of the rotation axis. Target voxel intensities are corrected for these number of occurrences; voxels not being hit stay blank.

### 3.3. Molecular ensembles for modelling disorder   

**Figure 2:**
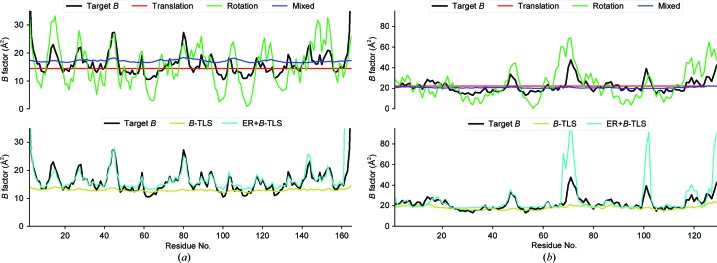
C^α^ *B*-factor traces of the disorder models used for diffuse scattering calculations: (*a*) for CypA and (*b*) for lysozyme. The top panels show the target *B*-­factor trace from the classically refined structures (Supplementary Table S1) and rigid-body models. The bottom panels show the target *B* factor, the *B*-­TLS subtracted before ensemble refinement and the final fluctuation from ensemble refinement recombined with the *B*-TLS (ER+*B*-TLS).

All calculations were performed with custom-made scripts using *cctbx* (Grosse-Kunstleve *et al.*, 2002 ▸). Four types of motion models, three rigid-body motion models and one rigid-body plus internal motion model, were generated for comparison with the measured and extracted experimental diffuse scattering. The three rigid-body-only models (Fig. 2 ▸, top panels) were fitted to the C^α^ *B*-factor fingerprint of the refined structure (target *B* in Fig. 2 ▸). Rotation angles were selected from a one-dimensional normal distribution, while translation vectors were extracted from a three-dimensional multivariate distribution. The rotation axis is a randomly generated vector. The variances of normal distribution, from which the rotation angles and translational displacements were generated, were fitted by a simplex minimization (*scitbx.simplex*) on the difference between the C^α^ *B*-factor trace and the *B* factors obtained from the root-mean-square fluctuation (r.m.s.f.) of 100 asymmetric units generated from the distributions. The disorder models then consist of 100 asymmetric units created with the fitted variances of either the translational distribution, the rotational distribution or a mixture of the two.

To model the internal motion of a protein in a crystal, ensemble refinement as implemented in *phenix.refine* (Burnley *et al.*, 2012 ▸; Adams *et al.*, 2010 ▸) was used. A parameter sweep over *p*_TLS_, *d*_TMP_ and τ_*x*_ was performed (Burnley *et al.*, 2012 ▸). The ensemble with the lowest *R*_free_ is chosen as the ‘best’ ensemble and used for further calculations (Supplementary Table S1). Before ensemble refinement is started, it is common practice to fit TLS matrices to the *B* factors of the input model (a refined crystal structure) and to subtract their contribution (*B*-TLS) from the *B*-factors columns. This prevents the refinement from sampling large-scale motion and forces the sampling of internal atomic fluctuations (Burnley *et al.*, 2012 ▸). For the diffuse scattering calculations presented here, these per-molecule TLS motions are reintroduced to the generated ensemble models. This is performed by fitting the rotation and translation variances to the C^α^ *B*-TLS trace found in the *B*-factor column of the ensemble models, similar to the method described above. The resulting translation and rotation operations are then randomly applied to asymmetric unit models from the ensemble refinement to create asymmetric units describing internal motion and *B*-TLS (Fig. 2 ▸, bottom panels).

As performed previously by Van Benschoten *et al.* (2015 ▸), we also calculated diffuse scattering from TLS models that were fitted to refined anisotropic displacement parameters *U*_*ij*_. The eigenvalues of (input *U*_*ij*_ − fitted *U*_*ij*_) were restricted to be positive. The **S**-matrix components were always set to zero. Fitted TLS matrices were used to generate ensembles of structures using *phenix.tls_as_xyz* (Urzhumtsev *et al.*, 2015 ▸).

### 3.4. Calculation of diffuse scattering from molecular ensembles   

We use supercells to sample diffuse scattering in reciprocal space in between the Bragg peaks at fractional Miller indices. The supercell crystals are of very limited size (5–10 unit cells in each dimension). However, all of the equations in Section 2 hold for these small crystals as long as *F_N_* = Ft[ρ(*r*)_*N*_] is calculated at (*h*_s_, *k*_s_, *l*_s_) values that are integer multiples of fractional (*h*, *k*, *l*). Otherwise shape transform ripples will dominate the diffuse pattern (Neder & Proffen, 2008 ▸), which does not occur in the observed diffraction patterns unless the crystal are truely nanometre-sized (Chapman *et al.*, 2011 ▸). Thus, we implement (9) by calculating the structure-factor variance of *N*_s_ supercells, 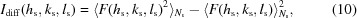


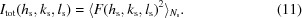



*N*_s_ is 100 throughout this paper. The asymmetric units describing the disorder are prepared for diffuse scattering calculation by setting all *B* factors to 0 and all occupancies to 1. Supercell parameters are chosen in such a way that the supercell crystals are close to cubic, and the smallest supercell is five unit cells in a row. This ensures that the reciprocal-space voxels in the final map will be close to cubic as well. Once the supercell dimensions have been chosen, the symmetry operations of the space group and unit-cell translations of the crystal are determined, forming a complete set of operations to fill the supercell. For each of the elements in the set, an asymmetric unit from the disorder model is chosen at random and the corresponding operation is applied. The supercell coordinate file, space group *P*1 and supercell size are passed on to *mmtbx.utils.fmodel_from_xray_structure* to be Fourier transformed to a resolution of 2 Å. A bulk-solvent model is used to represent the solvent. The structure factors and phases are written to a binary structure-factor file (.mtz). This is repeated 100 times in order to sample the full disorder that we want our supercells to represent. The process is performed in parallel using the *easy_mp* functionality in *cctbx*. 〈*F*(*h*_s_, *k*_s_, *l*_s_)〉^2^_100_ and 〈*F*(*h*_s_, *k*_s_, *l*_s_)^2^_100_〉 are then calculated, after which a final .mtz file is written containing the Miller indices from the supercell and the columns *I*_Bragg_, *I*_tot_ and *I*_diff_ (*I*_tot_ − *I*_Bragg_) that follow from (10) and (11).

The final diffuse intensities were placed in an array after applying Friedel symmetry to all supercell Miller indices. This array was written to a CCP4 .map-style file with supercell constants in Å^−1^ describing the reciprocal-space dimensions. No other symmetry operations were applied. The supercells are built with the space group of the crystals and thus the calculated diffuse maps should have the corresponding point-group symmetry.

For large supercells these calculations can become computationally intensive. For example, for the lysozyme diffuse scattering calculations discussed in this paper, the 5 × 5 × 10 supercell was *a* = *b* = 394.16, *c* = 382.32 Å, α = β = γ = 90.0°. This resulted in a supercell containing 250 unit cells, each filled with eight molecules made up of 1000 non-H atoms. The FFT resulted in a list of 15 550 023 Miller indices. The 100 temporary .mtz files took up 297 MB of disk space each and the final .mtz file was 356 MB in size. The map file used for further analysis had a file size of 230 MB.

### 3.5. Analysis of calculated diffuse scattering   

To compare experimental and model maps, the origins of the maps are aligned and a combined mask of unmeasured and noncalculated voxels is constructed. Noncalculated voxels in the model maps were set to 0. Calculated and experimental maps are scaled by their total unmasked intensities. The maps were displayed with *UCSF Chimera* (Pettersen *et al.*, 2004 ▸) for visual comparison. Linear correlation coefficients (CCs) between all unmasked points are calculated using *cctbx* *array_family flex.linear_correlation*. The correlation coefficients between voxels corresponding to the original Bragg reflections are calculated by masking the non-Bragg voxels.

Radially averaged intensities of the scaled maps are calculated by masking everything that is not within the resolution shell and calculating the mean in 20 resolution bins. Maps containing the radial average per voxel are constructed, saved and subtracted from the original maps. Correlation coefficients between these isotropic corrected maps are calculated similarly as above.

Scripts are available on GitHub (https://github.com/kroon-lab/scud).

## 4. Results   

### 4.1. Experimental diffuse maps   

The maps reconstructed from images as described in Section 3 have point-group symmetry 1 and are subsequently symmetrized using Friedel symmetry (linear correlation coefficient CC of 0.86 for CypA and 0.78 for lysozyme) or the Laue point group of the crystals, which is *mmm* for CypA (CC = 0.74) and 4/*mmm* for lysozyme (CC = 0.53). The diffuse maps for CypA [Figs. 1 ▸(*b*) and 1 ▸(*c*)] and lysozyme [Figs. 1 ▸(*e*) and 1 ▸(*f*)] viewed along the *l* axis (c*) in the −1 and the higher *mmm* and 4/*mmm* symmetries, respectively, show that in the lower point group the the noise level is quite high and averaging in *mmm* or 4/*mmm* improves the maps enormously. For lysozyme, Figs. 1 ▸(*e*) and 1 ▸(*f*) show that the fourfold symmetry is present in the lower point group. We verified that every target voxel (*h*_s_, *k*_s_, *l*_s_) was hit multiple times: for CypA the most frequent number of hits in a 9 × 8 × 5 oversampled map with point-group symmetry 1 was 44, but ranged from 0 to 507. Zero hits occur from detector-panel gaps, the beam-stop shadow and the cusp region of the rotation scan. For lysozyme, in the 5 × 5 × 10 oversampled map these values were 78 and 0–502. Voxel dimensions in the rotation direction (φ-range) are large in the case of wide slicing. We investigated what the consequence is for mapping into reciprocal space. When fine-slicing the lysozyme data at 0.3°, instead of at 1° as we used initially, the most frequent number of hits per target voxel increased to 100 and ranged between 0 and 1467, which implies that the subdivision of every voxel into 3.3 voxels does not generate 3.3 times the number of hits, and that many of them map to the same target voxel. The two maps look quite similar (CC = 0.62). The original data were fine-sliced to 0.1° but brought the diffuse scattering to the single-photon noise level and no good diffuse maps could be obtained. We conclude that a scan width of 0.5–1° is probably best for obtaining sufficient signal in the diffuse maps in the usual experimental setup at synchrotron beamlines. The subtraction of radial mean background intensity leads to negative pixel values in the diffuse maps. Chapman *et al.* (2017 ▸) have developed an improved method for background subtraction by using a discrete noisy Wilson distribution, by which average background intensities and their variance are determined. This method avoids the over-subtraction of background, while getting rid of almost all negative intensities. We did not correct the diffuse image intensities to obtain only positive intensities. The speckle structure, the distribution of intensities and linear correlation coefficient are not affected by the maps containing negative intensities.

We noticed that in projections of the complete three-dimensional diffuse maps intensities accumulated on the Bragg layers perpendicular to a* and b* in CypA and to c* in lysozyme (Supplementary Fig. S1). Such features could not be observed in individual slices as they are very weak. We confirmed that the kernel in our mode filter (21 × 21 pixels) was sufficiently large to not leave part of the Bragg spots behind (judged after mapping to three-dimensional reciprocal space), so we rule out these features being caused by Bragg peaks. Similar observations were made by Polikanov & Moore (2015 ▸). They found troughs between adjacent rows where the Bragg reflections were removed in diffuse patterns of ribosome. These features must be related to the lattice disorder rather than diffuse scattering caused by motion within the unit cell. Polikanov and Moore were able to reproduce this type of diffuse scattering using a model for acoustic displacement waves. By writing diffuse scattering in terms of structure-factor variances and structure-factor correlation coefficients between unit cells [which corresponds to our equation (7) and diffuse scattering of type (iii)], Moss *et al.* (2003 ▸) concluded that in soft molecular crystals the correlation coefficients fall off rapidly with **q**, the Brillouin zone vector, resulting in a broad acoustic peak at the Bragg positions. Such weak acoustic lattice vibrations must therefore be present in both CypA and lysozyme.

### 4.2. Calculated diffuse maps   

**Figure 3:**
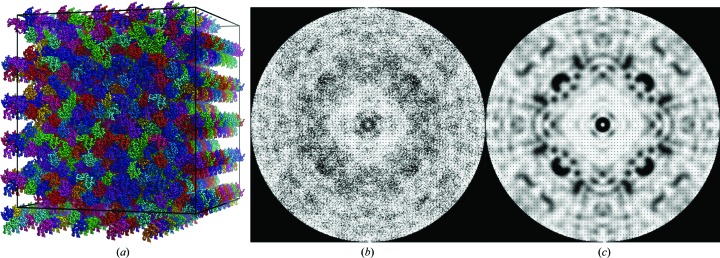
(*a*) 5 × 5 × 10 supercell of lysozyme molecules; the ‘mixed’ rigid-body disorder model was used to construct this supercell. (*b*) *F*(*h*_s_ *k*_s_0)^2^ slice of the Fourier transform of (*a*). (*c*) *I*_total_(*h*_s_ *k*_s_0) [= 〈*F*(*h*_s_ *k*_s_0)^2^〉] slice of diffuse scattering calculation from a ‘mixed’ rigid-body disorder model. 100 supercells have been constructed and the squared structure factors have been averaged. The diffuse features become more well defined. (*b*) and (*c*) are coloured from white (0) to black (75 × 10^6^).

Molecules (asymmetric units) randomly picked from the disorder models described previously were used to construct supercells [Fig. 3 ▸(*a*); Section 3]. The Fourier transforms of these supercells sample on and between the integer Miller indices of the original unit cell (Section 3). A Fourier transform of a single supercell [Fig. 3 ▸(*b*)] shows Bragg reflections of the original unit cell and a weak diffuse scattering pattern. When 100 supercells are Fourier transformed and the average total intensities are calculated, this results in well defined diffuse scattering under and between the Bragg reflections [Fig. 3 ▸(*c*)]. The Bragg reflections obey the symmetry and extinctions of the original space group (*P*4_3_2_1_2; see the systematic absences in the *h*_s_ = 0 and *k*_s_ = 0 directions). Diffuse scattering is calculated as the difference between the total scattering and the Bragg scattering.

### 4.3. Comparison of the diffuse scattering between models and data   

Linear correlation coefficients between all calculated maps and the data were calculated (Table 1 ▸; Section 3). For CypA, the modelled scattering from translational disorder has a correlation coefficient (CC) of 0.46 with the measured diffuse scattering; disorder modelled using a mix of translation and rotation gives a CC of 0.47 (Table 1 ▸). Van Benschoten *et al.* (2016 ▸) recorded the CypA data set and showed that diffuse scattering fitted by a liquid-like motion model resulted in a correlation coefficient of 0.518. However, the authors only compared the anisotropic components of both the measured and calculated diffuse scattering in their analysis. If we remove the isotropic components from the data (very little is left because of radially averaged background subtraction) and models, we obtain a CC of 0.51 for our translation-only model and a CC of 0.53 for a model from mixed rotation and translation, and thus we obtain comparable agreement.

For lysozyme, lower correlations between rigid-body models and the data were obtained than for CypA (CC = 0.29 for mixed translation and rotation). However, the agreement improves when considering only the diffuse scattering at the original Bragg positions (Table 1 ▸). The anisotropic components of the data and the calculated maps show an even better agreement: a CC of 0.45 for the mixed rigid-body disorder model.

The addition of internal motion to the rigid-body disorder models did not improve the correlation coefficients with the data. For CypA these correlation coefficients are comparable to those of rigid-body models (CC of 0.47 for Ensemble+*B*-TLS versus 0.47 for the mixed-disorder model), while for lysozyme the coefficients become worse. Modelled diffuse scattering maps show high correlation coefficients amongst each other (Table 1 ▸). The only exception is the poor resemblance of translation- and rotation-calculated maps (CC < 0.55), which is consistent with the findings of Moore (2009 ▸).

We generated an ensemble of molecules from refined TLS matrices, a method that was used previously by Van Benschoten *et al.* (2015 ▸), and calculated linear cross-correlations between the modelled scattering and the data. For CypA, CC_all_ and CC_aniso_ are 0.46 and 0.51, which are comparable to the translation CC values (CC_all_ of translation versus TLS of 0.93). For lysozyme, the CC with data for TLS models improved compared with translation models (CC_all_ = 0.33, CC_aniso_ = 0.37). This shows that the anisotropic translation matrix from the TLS model more accurately describes the true (anisotropic) translation behaviour (Supplementary Fig. S3).

## 5. Discussion   

**Figure 4:**
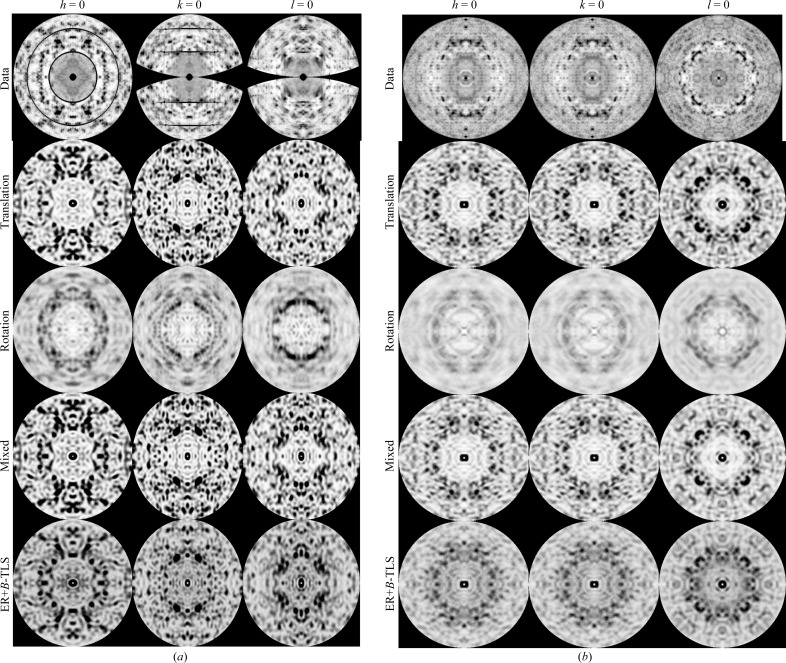
Slices through diffuse maps for (*a*) CypA and (*b*) lysozyme. Experimental data are coloured to obtain maximum contrast. We coloured the calculated translational diffuse map likewise; the other calculated maps are coloured on the same scale.

**Figure 5:**
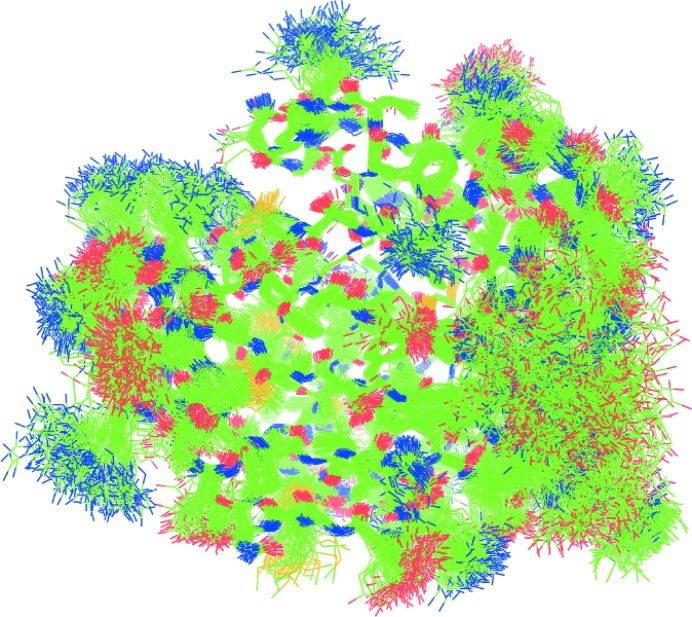
250 members of the ensemble for representing internal motion in CypA as obtained from ensemble refinement with *phenix.refine*. The structures are subsequently combined with rigid-body motion to calculate diffuse scattering maps.

Correlated motional disorder of atoms within the unit cells produces diffuse scattering of type (ii) (see Section 2). Such motions can be rigid-body movement of whole molecules or internal conformational mobility, or combinations thereof. We generated molecular models to describe such motions using the supercell method and calculated full oversampled three-dimensional diffuse maps. Diffuse maps from rigid-body models have a remarkable resemblance to experimental diffuse maps, as discussed below. Firstly, the linear correlation coefficients are comparable to those in earlier work by Van Benschoten *et al.* (2016 ▸) for CypA, but are lower for lysozyme. The latter is likely to be caused by the more noisy experimental data, as the CC between symmetrized and original maps is only 0.53 and fine- and wide-sliced data sets from the same image data produce maps with a CC of 0.62. Secondly, the two-dimensional zero zone slices (Fig. 4 ▸) and three-dimensional maps for both CypA and lysozyme (Supplementary Fig. S2) clearly show that throughout reciprocal space experimental diffuse features are reproduced by the mixed rigid-body models. Thirdly, the introduction of internal motion models in addition to rigid-body motions, which were obtained from ensemble refinement and were not specifically optimized to reproduce the diffuse scattering, does not improve the agreement (Table 1 ▸). Internal motions appear to only modulate the rigid-body diffuse scattering (compare the two lower rows in Fig. 4 ▸), although substantial motions occur (see, for example, the ensembles representing internal motions of CypA depicted in Fig. 5 ▸).

**Figure 6:**
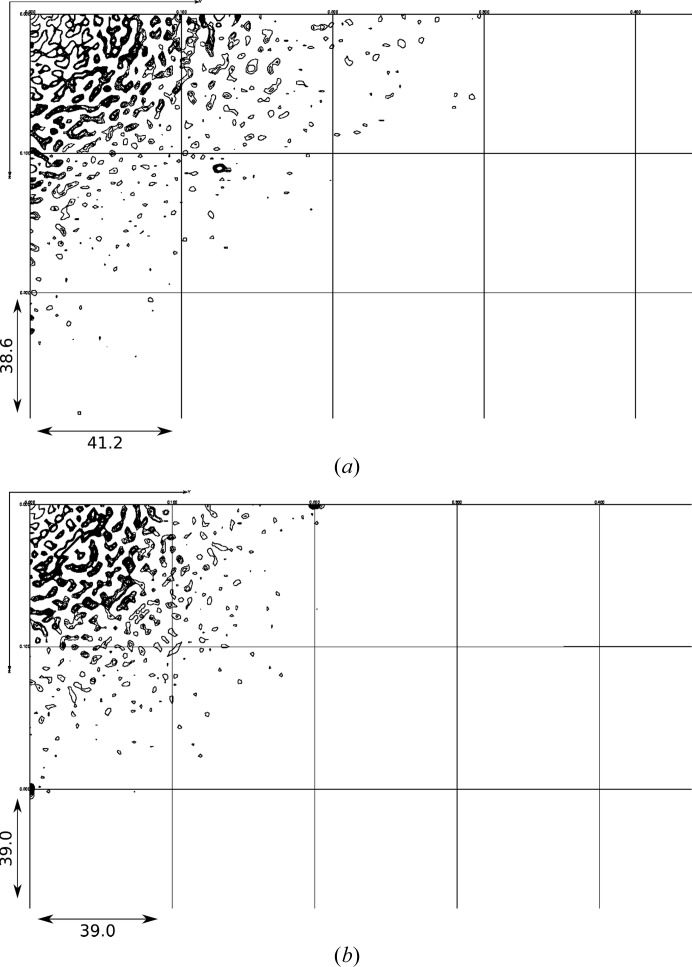
Fourier transform of experimental diffuse scattering intensities for (*a*) CypA and (*b*) lysozyme in the *ab* plane as calculated with the *CCP*4 FFT for Patterson module. Graphs were made with *MapSlicer* in *CCP*4. Gridlines are drawn at 1/10 of the supercell dimensions. Arrows indicate the size of one grid unit in Å.

The crystals considered here have a moderate degree of packing disorder (diffraction to 1.15 and 1.3 Å resolution for CypA and lysozyme) but are still sufficient to produce this type of diffuse scattering. Ayyer *et al.* (2016 ▸) and Chapman *et al.* (2017 ▸) observed continuous diffraction in the XFEL data of photosystem II (PSII) crystals that diffracted to only 4.5 Å resolution. They assumed this to be caused by translational displacements of individual molecules and showed that the total diffuse scattering is the incoherent sum of that of displaced symmetry-related molecules. This assumption allowed them to use oversampling techniques as practiced in coherent diffractive imaging and thereby to interatively phase to higher resolution than the Bragg diffraction. An unbiased estimation of the structural unit that is responsible for the continuous scattering was obtained from the size of the speckles in the diffraction pattern and its autocorrelation function, which indicates that for PSII this is a dimer. To verify our above results, we made such an independent estimation of the structural unit responsible for the diffuse scattering in CypA and lysozyme by calculating the autocorrelation function from our experimental diffuse maps. This is similar to calculating a Patterson map from Bragg data, as is common practice in crystallography. Indeed, we could feed the *CCP*4 Patterson module with our supercell (*h*_s_, *k*_s_, *k*_s_, *I*_diff_) array (Fig. 6 ▸). We found a size of 30–40 Å, corresponding to one molecule for both CypA and lysozyme, and consistent with our rigid-body models. A critical review (Wall *et al.*, 2018 ▸) questions the assumptions made by Ayyer and Chapman. We discuss some of the issues raised below.

(i) What effect does the presence of Bragg peaks have on phasing and resolution extension in the 4.5–3.5 Å range? Bragg reflections do not oversample reciprocal space so they would be hardly effective in iterative phasing.
(ii) Could the free-lunch effect be responsible for the phase improvement? The continuous diffraction beyond what Ayyer and coworkers call the Bragg limit is certainly not random; the speckle intensities are proportional to the incoherent sum of the squared Fourier transforms of the molecules and are quite strong because of the large displacements that cause the low Bragg limit and do provide useful information for phasing (see equation 5 and the discussion below it, and the role of the displacements **δ** in the strength of diffuse scattering). However, the success of the free-lunch approach can only be established by trying it.
(iii) Might an LLM model (or an ENM or an MD model) more accurately describe the diffuse scattering than the rigid-body translations of PSII dimers? Our results clearly show the dominant contributions of rigid-body translations. Most of the residue-wise *B* factor is caused by translation (the red base line in Fig. 2 ▸, for example, is 15 Å^2^ for CypA and 20 Å^2^ for lysozyme) and the variation from rigid-body rotations adds only a small portion, while internal motions occasionally add up to 10 Å^2^ (see the difference between the cyan and yellow curves in Fig. 2 ▸). We stress again that rigid-body translations are fully concerted motions and therefore their presence readily dominates the diffuse scattering.
(iv) Can the model be improved by assuming that the rigid units are coupled instead of independent? Such coupling between molecule motions would give rise to halos around the Bragg reflections, for which we indeed found evidence, but the signal is very weak (see Supplementary Fig. S1).
(v) Can the model be improved by including rigid-body rotations? Inspection of Fig. 4 ▸ shows that rotational diffuse scattering has a blurred appearence: the speckle structure fades at larger **Q**, much like the blurring in reciprocal space as carried out by Chapman *et al.* (2017 ▸). Moreover, the intensities in the simulated rotational diffuse maps are much lower, as can be understood by the smaller contribution of rotations to the r.m.s. deviations (the difference between the blue and red curves in Fig. 2 ▸ is the contribution of rotation to the *B* factors), as well as the fact that the atoms within the molecule do not all undergo the same displacements. Thus, our analysis indicates that assuming the main cause for continuous scattering to be translational rigid-body disorder is realistic. Although our mixed models are the best, the agreement with the translation-only disorder is large (CC > 0.92).

Our conclusions are different from previous work, where internal correlation motions were held to be responsible for diffuse scattering. LLM models for CypA (Van Benschoten *et al.*, 2015 ▸; Peck *et al.*, 2018 ▸) and tetragonal lysozyme (Clarage *et al.*, 1992 ▸) give fair agreement with diffuse scattering data, and likewise elastic network models for other protein crystals (Riccardi *et al.*, 2010 ▸). In both approaches the diffuse scattering is proportional to a convolution of the Fourier transform of the Patterson of the displaced structure and the Fourier transform of a displacement correlation function. This leads to speckles distributed over all of reciprocal space. The parameters in this model have been fitted to the diffuse scattering, and indeed its global appearance resembles that from the rigid-body translations (see Fig. 4 in Peck *et al.*, 2018 ▸). We have calculated from the Fourier transform of the exponential displacement correlation function that a correlation length of 7.1 Å, as Van Benschoten *et al.* (2016 ▸) found, leads to a speckle size of 1/33 Å^−1^, which is roughly in agreement with the size of the rigid unit as determined from the autocorrelation function of our diffuse data. In contrast, our ensemble structures that model internal correlated motions make only a small contribution to the diffuse scattering maps. Our models are from ensemble refinement of the Bragg data and are not fitted to correlated motion, so may not be fully representative, although we assume that the force field in the ensemble refinement ensures at least some correlated motions. Obviously, the motion that has the largest correlation between atoms is rigid-body translation, as all atoms move in a fully concerted manner, and therefore will always dominate the diffuse scattering (see equation 5 and the discussion below it). If only smaller structural units move in a correlated fashion the variances in structure factors are not that large (equation 8) and the diffuse intensities are much smaller. Molecular-dynamics simulations have been used to predict diffuse scattering with some success, especially since it was realized that long sampling times (>1 ns) were needed to reach convergence (Clarage *et al.*, 1995 ▸). Héry *et al.* (1998 ▸) concluded from MD simulations of one unit cell that in orthorhombic lysozyme crystals the molecules move only partially as rigid bodies, *i.e.* only the backbone atoms move as such. However, comparison with the data was only visual and on a single detector image. 10 ns MD simulations of the staphylococcal nuclease crystal by Meinhold & Smith (2005*a* ▸,*b* ▸) and subsequent principal component analysis (PCA) showed that the five lowest frequency large-amplitude components reproduce the main features of diffuse scattering. Whole-molecule motion was found to only represent part of the mean-square fluctuations, although these might be limited by periodic boundary conditions in the simulations. This restriction was overcome by Wall (2018 ▸) through MD simulations of 2 × 2 × 2 unit cells of the same protein. The agreement with diffuse scattering in terms of CC (0.68) is better than before. Unfortunately, limited insight is given into the three-dimensional diffuse maps as only one intersection with the Ewald sphere was shown and only averaged diffuse intensities in resolution shells. Furthermore, it is left unclear whether rigid-body translations occurred in the simulations, which is very possible because only unit-cell centre-of-mass translations were removed in the MD protocol, and with 32 molecules in the supercell there is plenty of room for relative motions of the molecules. In a recent paper, Peck *et al.* (2018 ▸) reanalysed the diffuse scattering of CypA using the same data that we used here and that was made public by Van Benschoten *et al.* (2016 ▸). Their conclusion is that intermolecular correlations are needed to explain the diffuse intensities that they extracted from the data. The analysis was based on a liquid-like motion model that was extended to include nearest-neighbour motional correlations. Although in the current paper we noted that evidence for longer range correlated motions is indeed found, we believe that their data actually still contain parts of the Bragg reflections and their large CC (0.71) can be attributed to these. Our diffuse maps look completely different, as we did not rely on predicted locations and the size of the Bragg reflections, but used mode filtering instead.

Simulated diffuse maps have an isotropic component that is part of the correlated motion, which we would prefer not to subtract. Clearly, the way we analysed the experimental data, by subtracting radially averaged background scattering, leads to the removal of all isotropic scattering, and as a consequence CC_aniso_ (Table 1 ▸) is larger than CC_all_. Improvements in this step of data processing in order to obtain better estimates of background scattering along the lines laid out by Chapman *et al.* (2017 ▸) will most likely give better agreement. One might question whether CC values in the range 0.45–0.6 are sufficient to conclude that any of the motion models are correct. We think that a large part of the disagreement comes from the noisy data and the processing methods. It is only after considering the features in full three-dimensional oversampled diffuse maps that we gained confidence in the validity of the rigid-body motion model.

We believe that our current approach by forward modelling of diffuse scattering in oversampled full three-dimensional reciprocal space, from well defined ensembles with translational, rotational and internal correlated motions, clearly shows the dominant influence of rigid-body translational disorder in protein crystals. Despite this, correlated internal motions could have an effect on the diffuse intensities. The challenge will be to model their weak contribution in order to reveal protein dynamics (Wall *et al.*, 2014 ▸). We are currently developing a supercell ensemble-refinement technique that uses the total scattering, *i.e.* Bragg intensities and diffuse scattered intensities. Realistic conformational motions, next to the rigid-body motions, can potentially be obtained from this kind of structural refinement.

## Supplementary Material

Supplementary Table and Figures.. DOI: 10.1107/S2052252519000927/cw5019sup1.pdf

## Figures and Tables

**Table 1:** Linear cross-correlation values between all models and data for CypA and lysozyme CC_all_ is calculated between all points in the map that are calculated or measured. CC_brg_ is calculated on positions corresponding to the integer Miller indices of the original unit cell. CC_aniso_ is calculated between all points in the maps that are measured or calculated after the isotropic component per resolution shell has been subtracted; for this, 20 resolution shells were used.

		Translation		Rotation		Mixed		Ensemble+*B*-TLS	
		CypA	Lysosyme	CypA	Lysosyme	CypA	Lysosyme	CypA	Lysosyme
Data	CC_all_	0.46	0.27	0.28	0.07	0.47	0.29	0.47	0.20
	CC_brg_	0.49	0.34	0.28	0.15	0.50	0.37	0.48	0.29
	CC_aniso_	0.51	0.43	0.36	0.39	0.53	0.45	0.52	0.45
Translation	CC_all_			0.47	0.55	0.95	0.93	0.79	0.74
	CC_brg_			0.43	0.56	0.94	0.92	0.78	0.76
	CC_aniso_			0.40	0.43	0.94	0.91	0.81	0.75
Rotation	CC_all_					0.51	0.55	0.48	0.60
	CC_brg_					0.48	0.54	0.46	0.60
	CC_aniso_					0.43	0.43	0.35	0.35
Mixed	CC_all_							0.82	0.72
	CC_brg_							0.82	0.71
	CC_aniso_							0.83	0.75

## Footnotes

1 Although the peak intensity as follows from equation (1) is proportional to *N* ^2^, after integration over the Bragg peak volume it is proportional to *N*.
