# Supplementary material for: Rigid-body motion is the main source of diffuse scattering in protein crystallography
Source: IUCrJ. 2019 Feb 16;6(Pt 2):277–89. doi: 10.1107/S2052252519000927 (PMC6400197; doi:10.1107/S2052252519000927)
Supplement: Supplementary file 1 [file m-06-00277-sup1.pdf]

IUCrJ

Volume 6 (2019)

Supporting information for article:

Rigid-body motion is the main source of diffuse scattering in protein crystallography

T. de Klijn, A. M. M. Schreurs and L. M. J. Kroon-Batenburg

Table S1. *Statistics for lysozyme and CypA data.*

	Lysozyme-RT-ID30A-3	CypA-RT-APS(PDB:5f66[1])
Cell Dimensions:		
a,b,c (Å)	78.83, 78.83, 38.23	42.91, 52.44, 89.12
α, β, γ (°)	90.0, 90.0, 90.0	90.0, 90.0, 90.0
**Data processing**		
Low resolution limit (Å)	27.87	44.56
High resolution limit (Å)	1.35 (1.3)	1.15(1.18)
Rmerge	0.126 (3.813)	0.12 (0.58)
Total number of observations	319933 (11324)	
Total number unique	27088 (4928)	68210
Mean((I)/sd(I))	7.94 (0.98)	9.9 (2.1)
CC(1/2)	0.998 (0.101)	
Completeness	100.0 (100.0)	94.8 (86.0)
Multiplicity	11.8 (6.6)	5.8 (5.0)
**Refinement**		
Resolution (Å)	1.4 - 27.87	1.15 - 44.56
Reflections used in refinement	24292 (2373)	68155 (6195)
R-free reflections	1219 (110)	3464 (313)
R-work	0.158 (0.290)	0.179 (0.358)
Rfree	0.165 (0.314)	0.183 (0.376)
Number of non-hydrogen atoms	1185	1446
Macromolecules	1115	1272
Ligand	2	-
Solvent	68	174
Protein Residues	129	164
RMS(bonds)	0.003	0.007
RMS(angles)	0.56	1.157
Ramachandran favored (%)	98.43	96.91
Ramachandran allowed (%)	1.57	3.09
Ramachandran outliers (%)	0.00	0.00
Rotamer outliers (%)	1.68	1.49
Clashscore	4.97	0.79
Average B-factor (Å^2)	26.05	21.42
Macromolecules	25.46	19.58
Ligands	47.74	-
Solvent	35.13	34.92
**Ensemble Refinement**		
τ_x (ps)	1.0	1.5
pTLS	0.2	0.2
dTMP	1.0	2.5
R-work	0.146	0.147
R-free	0.166	0.166
Number of models	250	250

[1] Data from (Van Benschoten *et al.*, 2016)

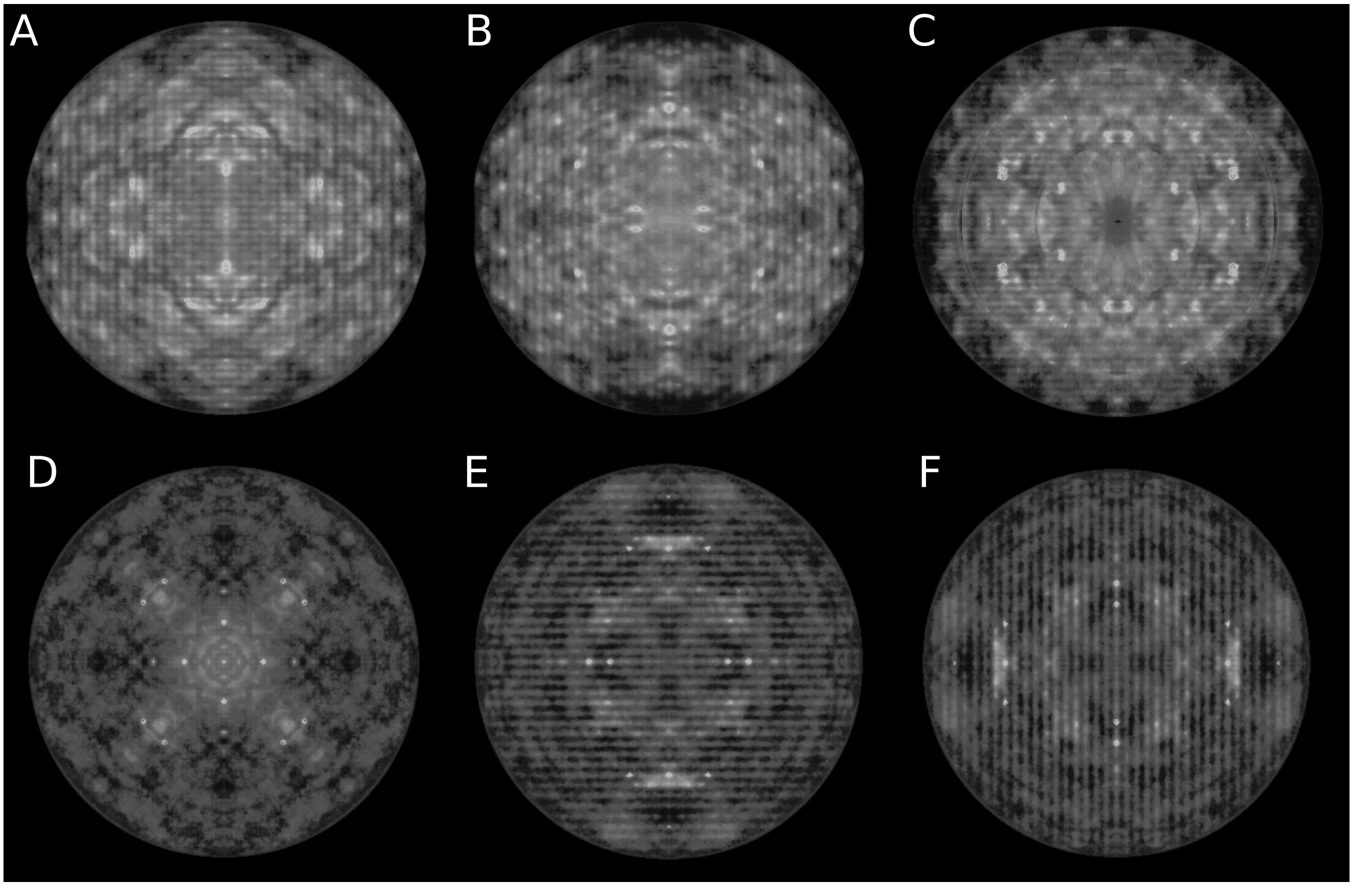

Fig. S1. Projections of 3D diffuse maps of CypA along $\mathbf{a}^*$,$\mathbf{b}^*$ and $\mathbf{c}^*$ **(A-C)** and lysozyme **(D-F)**.

Fig. S2. http://www.crystal.chem.uu.nl/diffusemaps. Sequential slices through maps along the l axis of data and the mixed models for both the CypA and the lysozyme data sets.

CypA:

$$T = \begin{bmatrix} 0.1189 & -0.0069 & -0.0054 \\ & 0.1220 & -0.0083 \\ & & 0.1240 \end{bmatrix}$$

$$L = \begin{bmatrix} 0.0148 & 0.0062 & -0.0119 \\ & 0.0288 & -0.0083 \\ & & 0.0112 \end{bmatrix}$$

Lysosyme:

$$T = \begin{bmatrix} 0.1135 & -0.1202 & -0.0093 \\ & 0.1007 & 0.0006 \\ & & 0.0668 \end{bmatrix}$$

$$L = \begin{bmatrix} 0.0000 & 0.0000 & 0.0000 \\ & 0.0000 & 0.0000 \\ & & 0.0000 \end{bmatrix}$$

Fig. S3. T and L matrices fitted to the refined B-factors of the CypA and lysozyme structures

References

Van Benschoten, A. H., Liu, L., Gonzalez, A., Brewster, A. S., Sauter, N. K., Fraser, J. S. & Wall, M. E. (2016). *Proc. Natl. Acad. Sci.* **113**(15), 201524048.
